# Dual pandemic of firearm injury and COVID-19 in Central and Southeastern Ohio: An interrupted time series analysis

**DOI:** 10.1371/journal.pone.0335813

**Published:** 2026-05-18

**Authors:** Hannah Williams, Megan Armstrong, Robin Alexander, Jonathan Groner, Bo Lu, Sherri Kovach, Roxanna Giambri, Henry Xiang

**Affiliations:** 1 Center for Pediatric Trauma Research, The Abigail Wexner Research Institute at Nationwide Children’s Hospital, Columbus, Ohio, United States of America; 2 Center for Injury Research and Policy, The Abigail Wexner Research Institute at Nationwide Children’s Hospital, Columbus, Ohio, United States of America; 3 Biostatistics Resource at Nationwide Children’s Hospital (BRANCH), The Ohio State University, Columbus, Ohio, United States of America; 4 Department of Surgery, The Ohio State University College of Medicine, Columbus, Ohio, United States of America; 5 Division of Biostatistics, The Ohio State University College of Public Health, Columbus, Ohio, United States of America; 6 COTS, Columbus, Ohio, United States of America; 7 Department of Pediatrics, The Ohio State University College of Medicine, Columbus, Ohio, United States of America; Facultad Latinoamericana de Ciencias Sociales Mexico, MEXICO

## Abstract

Firearm injuries increased as the United States faced the COVID-19 pandemic, a phenomenon some refer to as the “dual pandemic.” This study examines how one regional trauma system fared during the initial months of the dual pandemic and explores potential explanatory mechanisms of the surge of firearm injuries during the COVID-19 period. We used an interrupted time series model to compare quarterly data from 2016−2021 from the COTS (formerly known as the Central Ohio Trauma System) Regional Trauma Registry to examine the number of firearm injuries, mistriage rates of firearm-injured patients, injury intent, and cases with evidence of substance and alcohol abuse. Among 3,881 firearm-injured patients, demographic characteristics did not vary with respect to firearm injury, mistriage, mortality, substance use, or alcohol use. All but seven outcomes showed a significant level shift at the onset of the COVID-19 period, and all outcomes show a significant slope change during the COVID-19 period. All outcomes but one show a significant level shift at the end of the COVID-19 period, and all outcomes show a significant slope change from the conclusion of the COVID-19 period to the end of the study period. Wilcoxon ranked sum test shows no significant difference in the mean length of stay between the COVID-19 period and all other time included in the study period. This Midwest regional trauma system was affected by the dual pandemic of increased firearm injury during the COVID-19 lockdown. Findings highlight the roles of increased substance use and nonaccidental firearm injuries. Performance indicators reveal some evidence of strain within the region as the lockdown period progressed. Further research should identify region-specific causal mechanisms fueling the dual pandemic and compare the effects among urban, suburban, and rural communities.

## Introduction

The first two years of the COVID-19 pandemic aligned with a surge in firearm injuries nationwide [1–5], with 2021 holding the greatest amount of firearm injury-related deaths ever recorded [6]. In 2020 and 2021, trauma systems formed frontlines against both firearm injuries and COVID-19. The coinciding public health crises have been referred to as a “dual pandemic” [3]. The COVID-19 pandemic provided unique mechanisms with the possibility to contribute to increased firearm injury, including increased interpersonal violence and intentional self-harm alcohol and other substance use, and an influx of firearms into American households.

Disasters and mass traumatic events have been found to increase the burden of post-traumatic stress disorder [7] in communities. Both acute and prolonged situational stressors related to COVID-19 and the many stay-at-home orders have the potential to lead to PTSD and other mental and emotional sequelae [8–10]. While considering the complex relationship between mental illness and violence [11–13], the psychological impact of COVID-19 and lockdown periods combined with additional social and environmental stressors, could leave some individuals more vulnerable to engaging in violence against themselves or others. These phenomena are best understood through the lens of strain, in which negative stressors such as the loss of control and persistent grief of the pandemic, can lead some people to cope by engaging in criminalized behaviors [14].

The cumulative strains of the pandemic could reasonably have led to increased coping through substance use and resulting in associated firearm injuries. Substance use, particularly alcohol use, increases the odds of engaging in firearm-related behaviors, including firearm homicide and self-inflicted firearm injury [15–19]. The sharp increase in alcohol and other substance consumption during the pandemic [20–26] and the significant role of alcohol and other substances in firearm injuries [15–19] suggest the potential for an increase in alcohol-related firearm injuries treated by regional trauma systems in the early years of the pandemic. Appalachian regions of Southern and Southeastern Ohio are especially burdened by the opioid crisis [27], identifying the region as a potential hotbed for substance use-related firearm injury.

All of these challenges coincided with a time of increased firearm purchasing nationwide [28]. Around 3% of all American adults became first-time firearm owners from January 2019 to April 2021 and collectively exposed over 11 million people to household firearms [29], increasing rates of unintentional injury [30], suicide [31], and domestic homicide [32] into affected households [33]. The early months of the pandemic, specifically the lockdown period, saw a dramatic increase in firearm purchasing. From March to mid-July 2020, an estimated 6.5 million adults bought firearms for the first time [34]. The adverse effects of increased household gun exposure are particularly evident in the surge of intimate partner violence during stay-at-home-orders [35].

The initial wave of COVID-19 across the United States prompted widespread fear and anxiety over the ability of healthcare systems, particularly trauma teams and emergency departments, to withstand the threat of critically ill COVID-19 patients en masse. Stay-at-home and physical distancing orders prompted staffing policy shifts [36], potentially limiting the availability and overwhelming the workload capacity of trauma providers [37]. Healthcare workers were burnt out, and hospital resources had to be carefully rationed to avoid depletion [38,39]. Decreased hospital resource availability during the COVID-19 period was associated with higher mortality for both COVID-19 and non-COVID-19 patients [40–42].

Although all-cause mortality rates increased dramatically for hospitals nationwide during the COVID-19 period, trauma centers across different regions in the United States reported varying trauma activation rates [43,44], injury modalities, and trauma system utilization during the COVID-19 period. Causes of traumatic injury, diagnoses, and procedures were significantly changed by the pandemic [1,44–46]. Although heightened nationwide, crime records show that rates of firearm violence varied widely between states during the pandemic [47]. Trauma centers nationwide increased use of hospital resources to adjust accordingly to meet the changing demands associated with altered injury patterns [46].

Therefore, this study examines the response of one regional trauma system and explores some potential mechanisms of the “dual pandemic”. It aims to fill an important role by examining how one regional trauma system, COTS, fared under the weight of the dual pandemic and explores how contextual factors across Central, Southeast, and Southeast Central Ohio may have contributed to the local manifestation of the dual pandemic. Two specific objectives were: (1) to provide an epidemiological description of the dual pandemic of COVID-19 and firearm injury within a regional trauma system, and (2) to identify the effects of simultaneous public health crises on the quality of care delivered by a regional trauma system.

## Methods

COTS (formerly known as the Central Ohio Trauma System) includes two adult Level I trauma centers, one pediatric Level I trauma center, two adult Level II trauma centers, two adult Level III-N and three adult Level III trauma centers, as well as 34 acute care hospitals and 18 free-standing EDs across 37 of Ohio’s 88 counties. Its vast service area encompasses geographically, culturally, and politically diverse regions, including the urban center of Columbus and the surrounding Central Ohio metropolitan area, as well as rural communities in the Appalachian regions of Southeast Ohio. We obtained 2016–2021 regional trauma data from the COTS’s Trauma Registry, containing trauma-related pre-hospital, inpatient, and disposition data during the study period. The COTS trauma registry reporting follows the American College of Surgeons’ National Trauma Data Standard with minimal exceptions. The Institutional Review Board at Nationwide Children’s Hospital (IRB00000568) approved this study on June 6, 2022, and deemed the study ‘No more than minimal risk’ Human Subjects’ Research. Data were accessed for research purposes beginning on January 6, 2023. The COTS trauma registry contains fully deidentified data.

This retrospective, cross-sectional study used a segmented interrupted time series model (ITS) [48] to analyze quarterly (Q) data following the COVID-19 lockdown among firearm-related injury patients treated within COTS from January 1, 2016, to December 31, 2021, using the International Classification of Diseases, Tenth Revision (ICD-10) code for firearm-related injuries. Any injuries from air guns, gas or spring guns, or pellet guns were excluded. Specific outcomes include the number of total trauma activations, the number of firearm injuries, over- and undertriage rates, mortality rates (limited to deaths occurring in the ED or hospital), a breakdown by injury intent (assault, intentional self-harm, legal intervention, unintentional, and undetermined), and the number of cases with clinical evidence of substance and alcohol use. We used mistriage, both under and overtriage, and case mortality as measures of trauma system performance. Undertriage refers to instances in which the trauma alert did not capture the severity of the injury among those patients with an injury severity score (ISS) greater than or equal to 10. Overtriage refers to the instances in which the trauma alert called indicated more severe injuries than were present in patients with an ISS less than 10. Mistriage rates were calculated using the Cribari Matrix [49]. We defined the COVID-19 lockdown period as March 16, 2020, to December 20, 2020, to reflect Ohio’s stay-at-home orders and phased reopening [50,51]. This study used quarterly indicators to account for trauma seasonality. The interrupted time series models were conducted in R (version 5), and plots were created using the ggplot2 package. Quarterly comparisons were determined using SPSS Statistics Version: 29.0.0.0 (241). Additionally, this study used a Wilcoxon rank sum test to determine if the mean length of stay differed between the COVID-19 lockdown period and all of the time before and after the lockdown period that was included in this study.

We hypothesized the number of firearm injuries to undergo a level increase and a rising slope change that continues past the lockdown period, as evidence of the dual pandemic in the region. We hypothesized that performance indicators (undertriage and mortality rate) would experience temporary level and slope decreases during the lockdown-period as trauma centers become increasingly strained. At the same time, we hypothesized that intentional self-harm injuries experienced level and slope increases to reflect increased suicidality during the lockdown period that return to normal levels after the lockdown period. Finally, we hypothesized that increased maladaptive coping would lead to a level increase of clinical evidence of substance and/or alcohol use among those presenting with firearm injuries with continuously heightened rates continuing to the remainder of 2020 and beyond, exhibiting level and slope increases beginning, during, and after the lockdown period.

## Results

**Fig 1:**
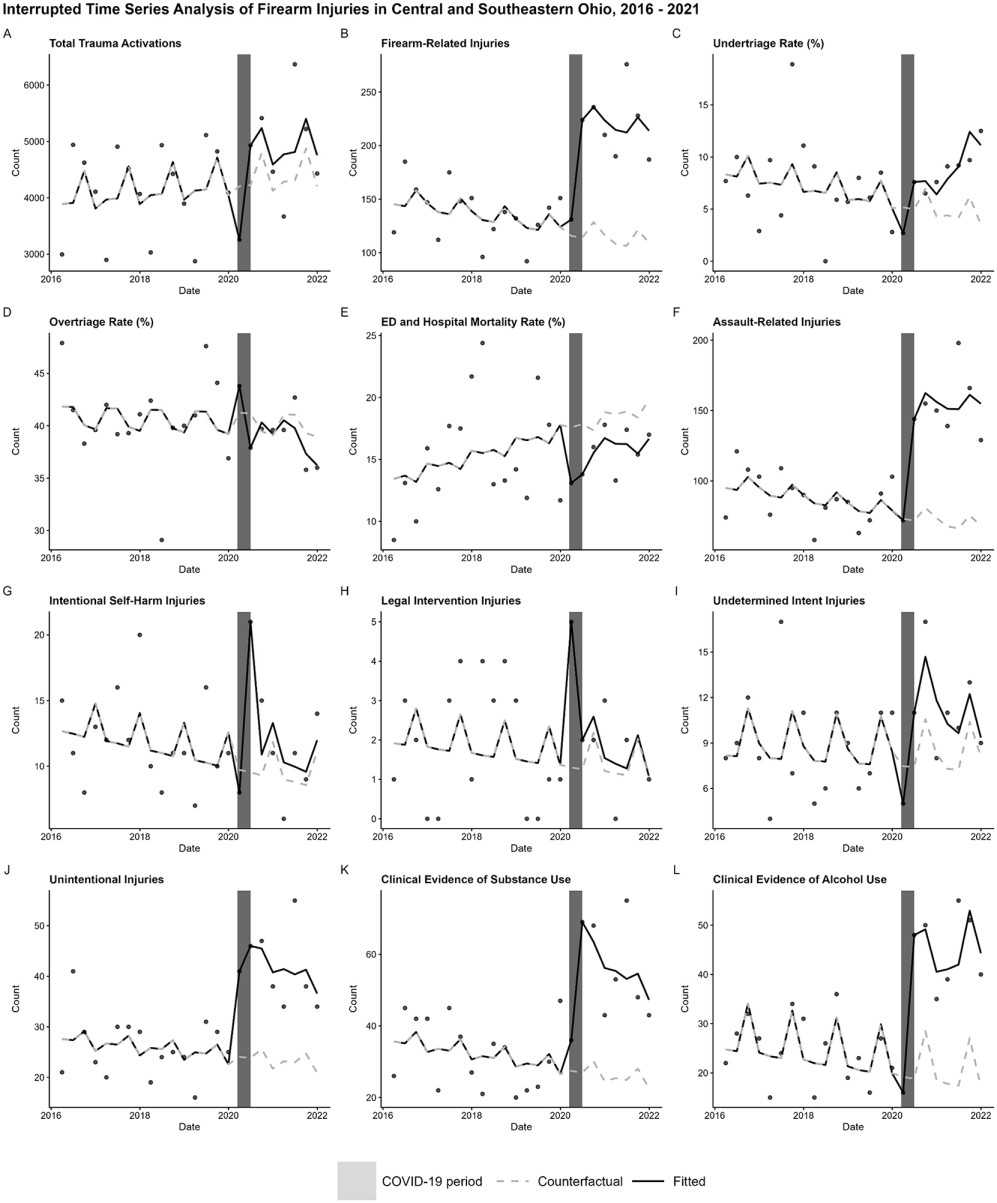
Interrupted time series analysis of quarterly trauma-related outcomes before, during, and after the COVID lockdown period (2016-2023).

A total of 3,881 cases of firearm injury were included. There were no significant effects between the COVID-19 period and age group, sex, race, ethnicity, or primary payer within the entire sample. Additionally, there were no significant effects of age group, sex, race, ethnicity, or primary payer on the case mortality rate or the undertriage rate among those with an Injury Severity Score (ISS) greater than 10. All but seven outcomes showed a significant level shift at the onset of the COVID-19 period (Table 1; Fig 1). Those without significant variation from the counterfactual include total firearm injuries (Estimate: 15.34; 95% CI: −4.85, 35.52; p = 0.156), undertriage rate (−2.48; −5.54, 0.59; p = 0.156), overtriage rate (2.65; −3.41, 8.54; p = 0.413), intentional self-harm injuries (−1.72; −3.58, 0.13; p = 0.088), clinical evidence of substance use (8.55; −1.32, 18.41; p = 0.109), and clinical evidence of alcohol use (−3.18; −6.67, 0.31; p = 0.093). All outcomes show a significant slope change during the COVID-19 period (Table 1; Fig 1). All outcomes show a significant level shift at the end of the COVID-19 period (Table 1; Fig 1) with the exception of mortality rate (−2.70; −5.95, 0.55; p = 0.123). All outcomes show a significant slope change from the conclusion of the COVID-19 period to the end of the study period (Table 1; Fig 1). Analysis of the Autocorrelation Function (ACF) and Partial Autocorrelation Function (PACF) series residuals showed no evidence of autocorrelation that could obscure ITS results. Wilcoxon ranked sum test shows no significant difference in the mean length of stay between the COVID-19 period (n = 644) and all of the time before and after the lockdown period that was included in this study (n = 3190) (Z = −1.09; p = 0.28).

**Table 1:** Interrupted time series analysis of quarterly trauma-related outcomes before, during, and after the COVID lockdown period (2016-2023).

Outcome	Term	Estimate (95% CI)	P value
Total trauma activations	COVID-period level shift	−950.42 (−1343.04, −557.81)	<0.001
	COVID-period slope change	1654.19 (1622.62, 1685.76)	<0.001
	Post-COVID level shift	−2865.50 (−3223.27, −2507.74)	<0.001
	Post-COVID slope change	−1632.92 (−1703.12, −1562.72)	<0.001
Total firearm injuries	COVID-period level shift	15.34 (−4.85, 35.52)	0.156
	COVID-period slope change	94.85 (93.47, 96.24)	<0.001
	Post-COVID level shift	−82.15 (−97.99, −66.31)	<0.001
	Post-COVID slope change	−95.42 (−99.40, −91.45)	<0.001
Undertriage rate (%)	COVID-period level shift	−2.48 (−5.54, 0.59)	0.133
	COVID-period slope change	5.10 (4.83, 5.37)	<0.001
	Post-COVID level shift	−9.46 (−11.61, −7.30)	<0.001
	Post-COVID slope change	−3.72 (−4.15, −3.28)	<0.001
Overtriage rate (%)	COVID-period level shift	2.56 (−3.41, 8.54)	0.413
	COVID-period slope change	−5.86 (−6.28, −5.44)	<0.001
	Post-COVID level shift	12.58 (7.79, 17.37)	<0.001
	Post-COVID slope change	5.15 (4.82, 5.49)	<0.001
ED & hospital mortality rate (%)	COVID-period level shift	−4.49 (−7.28, −1.71)	0.006
	COVID-period slope change	0.44 (0.09, 0.79)	0.027
	Post-COVID level shift	−2.70 (−5.95, 0.55)	0.123
	Post-COVID slope change	−0.72 (−0.99, −0.44)	<0.001
Injury intent: Assault	COVID-period level shift	−1.11 (−14.36, 12.13)	0.871
	COVID-period slope change	73.37 (72.43, 74.30)	<0.001
	Post-COVID level shift	−65.19 (−75.52, −54.87)	<0.001
	Post-COVID slope change	−72.32 (−75.26, −69.37)	<0.001
Injury intent: Intentional self-harm	COVID-period level shift	−1.72 (−3.58, 0.13)	0.088
	COVID-period slope change	13.18 (13.05, 13.32)	<0.001
	Post-COVID level shift	−24.78 (−27.14, −22.41)	<0.001
	Post-COVID slope change	−13.33 (−13.96, −12.70)	<0.001
Injury intent: Legal intervention*	COVID-period level shift	3.70 (1.51, 5.89)	0.004
	COVID-period slope change	−2.96 (−3.12, −2.80)	<0.001
	Post-COVID level shift	6.33 (4.37, 8.30)	<0.001
	Post-COVID slope change	2.88 (2.71, 3.05)	<0.001
Injury intent: Undetermined	COVID-period level shift	−2.47 (−4.41, −0.53)	0.024
	COVID-period slope change	6.04 (5.92, 6.17)	<0.001
	Post-COVID level shift	−7.98 (−10.09, −5.86)	<0.001
	Post-COVID slope change	−6.61 (−7.03, −6.20)	<0.001
Injury intent: Unintentional	COVID-period level shift	16.94 (12.49, 21.39)	<0.001
	COVID-period slope change	5.22 (4.90, 5.54)	<0.001
	Post-COVID level shift	9.46 (6.15, 12.78)	<0.001
	Post-COVID slope change	−6.05 (−6.67, −5.42)	<0.001
Clinical evidence of substance use	COVID-period level shift	8.55 (−1.32, 18.41)	0.109
	COVID-period slope change	33.51 (32.77, 34.26)	<0.001
	Post-COVID level shift	−33.66 (−41.26, −26.05)	<0.001
	Post-COVID slope change	−35.22 (−36.54, −33.91)	<0.001
Clinical evidence of alcohol use	COVID-period level shift	−3.18 (−6.67, 0.31)	0.093
	COVID-period slope change	32.35 (32.17, 32.52)	<0.001
	Post-COVID level shift	−44.02 (−46.68, −41.37)	<0.001
	Post-COVID slope change	−31.05 (−32.04, −30.06)	<0.001

* Over the entire study period, no quarter exceeded 5 injuries related to legal intervention (median = 2).

## Discussion

Findings show that Central, Southern, and Southeastern Ohio did indeed experience a sharp uptick in firearm injuries presenting to trauma centers during the COVID-19 lockdown period. While the immediate level shift was not significant, the lockdown period showed a continually and swiftly rising trend of increased firearm injury. This spike was followed by an immediate significant decline at the end of the lockdown period which continued until the end of the study period. The pattern observed in overall firearm injuries shows that while an increase did not immediately coincide with statewide stay-at-home orders, once they had been established, Ohioans were presenting to trauma centers with firearm injuries at increasingly rising rates. The lagged effect from the beginning of the lockdown period until rising rates of firearm injury may be attributed to the significant level decrease in the total number of trauma activations as the initial shock of the stay-at-home-order caused emergency department usage to drop [52]. This lag may also be explained by a short-lived immediate decrease in interpersonal violence exposure as individuals stepped away from their social circles before the COVID era brought significantly increased relative risk of firearm violence [47]. For firearm injuries, the significantly increased slope change during the lockdown period, decreased level shift at the end of the lockdown period, and decreased slope change after the lockdown period reflect the trends seen in the number of total trauma activations. The results for the total number of firearm injuries reflect the estimated effects of stay-at-home orders in other parts of the United States [53].

The regional trauma system did show some evidence of strain during the time of increased firearm injury during the first stages of widespread COVID-19 infection. Undertriage rates mirrored the pattern of overall firearm injury including the initial lag at the onset of the stay-at-home order. As the lockdown occurred, rates of undertriage among firearm-injured patients rapidly rose. Across COTS overall, there is evidence that trauma teams failed to initiate the appropriate trauma activation levels for critically firearm-injured patients. Overtriage did not experience a level shift at the onset of the lockdown and exhibited significant decline during the lockdown period, which would align with theories of resource strain among affected trauma centers [54]. There may have been some overcorrection to the rising rates of undertriage, as overtriage rates climbed, and undertriage rates dropped after the lockdown period.

The mortality rate among firearm-injured patients exhibited an immediate level decrease at the start of the lockdown period, again presenting some evidence of an initial shock of stay-at-home orders. This was followed by a significantly increasing trend during the lockdown period that reflects the rise in firearm injuries overall. Using mistriage as a measure of performance, the pattern observed for the mortality rate supports the idea that trauma centers across the region were negatively affected by the dual pandemic. Additionally, mortality did not exhibit an immediate level shift at the end of the lockdown period while the slope decreased significantly from the end of the lockdown period to the end of the study period. This may indicate that the trauma system gradually recovered from the impacts of the lockdown period, as the stressors of the dual pandemic had a lasting effect. Results echo the findings of a nationally representative sample [55].

Assault and intentional self-harm by firearm followed the same pattern as the overall trend. This suggests that intentional harm by firearm, including suicide and interpersonal violence, contributed to the manifestation of the dual pandemic in this region. Similar studies found that firearm injury with intent of assault spiked with the onset of the lockdown period [1], especially among pediatric populations [56]. The delayed presentation of increased mortality overall, increased firearm injury among intentional injuries, injuries of undetermined intent, and evidence of substance and alcohol use could highlight the initial shock of the lockdown, as individuals’ routines were immediately interrupted. The sharp increase during the lockdown period reveals that it did not take long for Ohioans to experience increased exposure to the stressors of lockdown that could lead to increased firearm injury such as mental health challenges and maladaptive coping.

Unintentional injuries, however, did see an immediate increase that continued through the end of the lockdown period before their eventual decline after the lockdown had ended. This could be explained by the fact that people were spending more time where guns were stored, as school-aged children [57] and nonessential workers were present in the home for longer durations than before the lockdown. The immediate level shift at both the beginning and end of the lockdown period shows the pervasiveness of unintentional firearm injuries in the region during this time. Injuries due to legal intervention experienced an increase at the beginning of the lockdown period followed by a decreasing slope change during the lockdown period. Both the level and slope increased after the lockdown period, which coincides with heightened sociopolitical violence involving law enforcement during that time [58]. However, the number of injuries related to injury intent per quarter are too small to meaningfully interpret results of the corresponding ITS model.

Overall, results from this study are mixed. While the significantly increased slope during the lockdown period was evidence of the dual pandemic in the region, firearm injuries did not remain heightened after the lockdown period, as predicted. The temporary slope increase of performance indicators (undertriage and mortality rate) reveals evidence of strain among the region’s trauma centers. Findings support this study’s hypotheses regarding an increase in intentional self-harm firearm injuries during and after the lockdown period, with assault injuries following the same pattern. Unintentional injuries experience an immediate and sustained increase that did not return until a gradual decline after the lockdown period. While heightened substance and alcohol use among those with firearm injuries increased during the lockdown period as expected, they did not remain heightened after the lockdown period had ended. Non-significant findings for the initial level shift among most variables revealed a lag in presentation of firearm injuries that indicates an initial shock of stay-at-home orders before the manifestation of the dual pandemic, and the null post-COVID level shift in mortality suggests a gradual recovery as the lockdown period ended.

## Limitations

Although regional data is a key factor of this study, it prevents generalization of our findings to other populations. Additionally, data lacked location information, preventing any analyses of urbanicity/rurality, which would be valuable considering the higher COVID-19 case and mortality rates among rural areas [59] such as the Appalachian communities included in this study. The cross-sectional identity of these data prevents causal claims. Registry data is subject to variability, considering the large area and variety of institutions serviced by COTS.

## Conclusions

This study analyzed the dual pandemic of COVID-19 and firearm injury at the regional level using data from the area’s trauma centers. Results show that Central, Southern, and Southeastern Ohio experienced the dual pandemic and highlight the roles of increased substance use and nonaccidental firearm injuries, such as assault and suicide. Increased rates of undertriage paired with increased mortality among firearm injuries exhibit the strain experienced by trauma centers in the region. Further research is necessary to identify region-specific causal mechanisms fueling the dual pandemic and to compare the effects among urban, suburban, and rural communities.
